# Potential effects of polychlorinated biphenyls (PCBs) and selected organochlorine pesticides (OCPs) on immune cells and blood biochemistry measures: a cross-sectional assessment of the NHANES 2003-2004 data

**DOI:** 10.1186/1476-069X-13-114

**Published:** 2014-12-16

**Authors:** Berrin Serdar, William G LeBlanc, Jill M Norris, L Miriam Dickinson

**Affiliations:** Environmental and Occupational Health Department, Colorado School of Public Health, University of Colorado Denver, Colorado, Denver USA; Department of Family Medicine, School of Medicine, University of Colorado Denver, Colorado, Denver USA; Epidemiology Department, Colorado School of Public Health, University of Colorado Denver, Colorado, Denver USA

**Keywords:** PCBs, Organochlorine pesticides, Immunotoxicity, Blood count, NHANES

## Abstract

**Background:**

Polychlorinated biphenyls (PCBs) and organochlorine pesticides (OCPs) are widely distributed in the environment and may have adverse effects on the immune system.

**Methods:**

Lipid adjusted serum levels of 19 Dioxin Like (DL), 17 Non Dioxin Like (NDL) PCBs, 5 OCPs, and measures of complete blood count and routine biochemistry profile were obtained from the NHANES 2003-2004 cycle. For each of the PCB/OCP variables, individuals were put into four exposure groups and blood markers were compared across these groups.

**Results:**

Serum levels of PCBs and OCPs increased with age. Total white blood cell (WBC) count, red blood cells (RBC), hemoglobin, and hematocrit measures were lowest in the group with the highest serum PCBs. Results for the OCPs varied. For Mirex, WBC declined in the highest exposure; no significant differences were observed for p-p’-DDT or p-p’-DDE; and higher levels of WBC were observed at the highest exposure groups of serum *trans*-nonachlor and oxychlordane. Liver enzymes (AST, ALT, and GGT) were significantly higher in the highest exposure groups of PCBs/OCPs.

**Conclusions:**

We observed significant associations between PCB/OCP levels and blood markers in the general population. All of the levels were within normal ranges but the consistency of results is remarkable and may reflect subclinical effects. Largest differences were observed for NDL PCBs. Thus, routine application of toxic equivalency factors, which assume dioxin like mechanisms and aryl hydrocarbon receptor involvement, may not adequately reflect the effects of NDL PCBs in the mixture.

**Electronic supplementary material:**

The online version of this article (doi:10.1186/1476-069X-13-114) contains supplementary material, which is available to authorized users.

## Background

Polychlorinated biphenyls (PCBs) and organochlorine pesticides (OCPs) are persistent organic pollutants that have been linked to many health concerns involving the liver, skin, reproductive, endocrine, neurological and immune system [1–4]. PCBs have recently been classified as carcinogenic to humans (Group 1) by the IARC [5], while some OCPs, such as chlordane and DDT (and its derivatives DDE and DDD), have been grouped as possible human carcinogens (Group 2B) [6]. Following their industrial introduction in 1920’s, PCBs were widely used in the U.S. as coolants and lubricants in transformers, capacitors, and other electrical equipment until late 1970s when their manufacture was banned [1]. Human exposure to PCBs usually involves a mixture of different congeners and happens through contaminated air, water, or food. PCBs and OCPs accumulate in the food chain and higher exposures in the general population are among those who frequently consume contaminated meat, fish and poultry. The estimated elimination half-lives of PCBs vary by congener type and range between 1.4 years for PCB28 to 15.5 years for PCB170 [7]. Higher chlorinated PCBs are thought to be more persistent in the environment, more resistant to biotransformation, and stay longer in the body [7, 8]. Recently, an argument was made that the positions of chlorine atoms and not their total number is more predictive for biotransformation and elimination [8].

The human immune system is highly sensitive to toxic effects but little is known of the long term effects of low exposures to PCBs or OCPs in the general population. Recent reports raised concerns over immune effects of chlorinated pollutants [9–12]. Experimental animal studies and in vitro assays with a few selected PCBs show immunosuppressive effects of PCBs [12–19]. Similarly, accidental exposures to high levels of PCBs resulted in immunosuppressive effects in humans [20]. Among the different testing schemes recommended to assess immunotoxicity in humans, the most common tests involve routine hematologic parameters, such as blood counts with differentials, serum clinical chemistry measures, and more specific tests evaluating cellular and humoral immune response [21]. Total white blood cell (WBC) count has been considered as a useful marker of immune changes in humans, with absolute numbers providing more reliable information than percentages [21]. The main objective of this study is to examine possible associations between blood levels of selected PCBs, organochlorine pesticides and common hematologic and immune markers, such as blood count and blood biochemistry data in a group representative of the United States general population using data of the National Health Examination and Survey 2003-2004.

## Methods

### Study design

The National Health and Nutrition Examination Survey (NHANES) is an ongoing cross-sectional survey designed to be nationally representative of the non-institutionalized U.S. civilian population conducted annually since 1999 by the Centers for Disease Control and Prevention (CDC). It aims to evaluate the health and nutritional status of adults and children in the United States and the survey combines interviews, physical examinations and laboratory data.

### Biomarkers of exposure

Blood serum concentrations of PCBs and organochlorine pesticides were measured in a representative, random one-third subsample of people aged 12 years and older. Polychlorinated dibenzo-p-dioxins, dibenzofurans, and non-ortho substituted or coplanar polychlorinated biphenyls were quantified by high-resolution gas chromatography/isotope-dilution high-resolution mass spectrometry. Detection limits (LOD) varied by PCB congener and values below LOD were replaced with LOD divided by the square root of 2. More detailed descriptions of analytical methods have been reported previously [22–24]. Lipid-adjusted serum concentrations (ng/g unless stated otherwise) for individual PCB congeners, the sum of dioxin-like PCB congeners (DL PCBs) and the sum of non-dioxin like PCB congeners (NDL PCBs) were used in this study. Since analytical detection limits of serum PCBs varied greatly in previous cycles, all analyses were limited to the most recent 2003-2004 survey cycle. Chemicals with more than 90% below detection limit values were excluded from analyses. The group of DL PCBs was a combination of dioxin like chemicals such as polychlorinated dibenzofurans, polychlorinated dibenzo-p-dioxins, coplanar polychlorinated biphenyls, and mono-ortho-substituted polychlorinated biphenyls. The total of 19 DL PCBs included: 105; 118; 126; 156; 157; 167; 169; 1,2,3,7,8-pentachlorodibenzo-p-dioxin (D01, pg/g); 1,2,3,6,7,8-hexachlorodibenzo-p-dioxin (D03, pg/g); 1,2,3,7,8,9- hexachlorodibenzo-p-dioxin (D04, pg/g); 1,2,3,4,6,7,8-heptachlorodibenzo-p-dioxin (D05, pg/g); 1,2,3,4,6,7,8,9-octachlorodibenzo-p-dioxin (D07, pg/g); 2,3,4,7,8-pentachlorodibenzofuran (F03, pg/g); 1,2,3,4,7,8-hexachlorodibenzofuran (F04, pg/g); 1,2,3,6,7,8-hexachlorodibenzofuran (F05, pg/g); 1,2,3,4,6,7,8-heptachlorodibenzofuran (F08, pg/g); 1,2,3,4,6,7,8,9-octachlorodibenzofuran (F10, pg/g); 3,4,4',5-tetrachlorobiphenyl (TC2, pg/g); 2,3,7,8-tetrachlorodibenzo-p-dioxin (TCDD, pg/g). The following congeners were included in the total of 17 NDL PCBs: 28; 52; 66; 74; 99; 101; 128; 138; 146; 153; 170; 172; 177; 178; 180; 183; 187. Analyses of the OCPs included lipid adjusted (ng/g) serum values of the following pesticides: p-p’-DDE; p-p’-DDT, mirex, trans-nonachlor, and oxychlordane.

### Blood count and biochemistry data

Complete blood count (CBC) measurements with 5-part differential in whole blood were obtained based on the Beckman Coulter method of counting and sizing, in combination with an automatic diluting and mixing device for sample processing, and a single beam photometer for hemoglobinometry as described previously [25]. Routine biochemistry profile analysis was performed using Beckman Synchron LX20 analyzer [25]. Values of CBC and differential count included the following measures: white blood cell count, red blood cell count, platelet count, basophils number, eosinophils number, lymphocyte number, monocyte number, segmented neutrophils number, basophils %, eosinophils %, lymphocyte %, monocyte %, segmented neutrophils %, hemoglobin concentration, hematocrit, mean cell volume, mean cell hemoglobin, and mean cell hemoglobin concentration. Biochemistry profiles included all routine measurements, except for measures of triglycerides and cholesterol. These blood lipid values were excluded from analyses, since all chlorinated chemical biomarker levels were adjusted for total lipids that were calculated based on blood triglyceride and cholesterol levels. The biochemistry variables included: albumin, alanine aminotransferase (ALT), aspartate aminotransferase (AST), alkaline phosphatase, blood urea nitrogen, total calcium, bicarbonate, gamma glutamyl transferase (GGT), glucose, iron, lactate dehydrogenase (LDH), phosphorus, bilirubin, total protein, uric acid, creatinine, sodium, potassium, chloride, osmolality, and globulin. A detailed list of these variables is provided elsewhere [26, 27]. Additionally, level of serum C-Reactive Protein was evaluated as a dependent variable.

### Statistical analyses

The NHANES 2003-2004 data for lipid adjusted PCBs and OCPs (for individuals aged 12 and older), complete blood count data and routine biochemistry profile analyses (see above for complete list) were used in this study. All statistical analyses were conducted using SAS system version 9.4 (Cary, NC) adjusting for relevant survey design, subsample, and population weights. Age and gender are common factors influencing hematologic and immunity markers in the general population [21, 28] and thus have been considered as potential confounders in all comparisons.

For each of the 41 PCB/OCP variables, an indicator variable (41 total) was created to place individuals into one of four exposure groups (“pseudo-quartiles”) by exposure level to that compound. The first category for every indicator variable contained all individuals with a “below limit of detection (LOD)” measurement for that respective compound. In six cases (see below for list) there were no individuals with a below LOD measurement. If there were fewer than 100 individuals below LOD for a compound, the sample was simply split into quartiles of approximately 450 individuals per quartile. In these cases, individuals with below LOD measurements were included I the exposure group of individuals with the lowest measureable quantities of the PCB/OCP compound. This occurred for 15 PCBs and one OCP. If there were 100 or more individuals below LOD, they alone formed the first exposure group, i.e., only below LOD individuals were in that group. This occurred for 21 PCBs and 4 OCPs. In summary, each individual had 41 indicator variables, one for each PCB, OCP, which ranged in value from 0 to 3, with 0 indicating the lowest level of exposure to a particular compound.

Composition of the exposure groups of PCB/OCP variables were as follows: Six PCB variables with no below LOD values: 28; 52; 74; 118; 138; and 153. Nine PCB variables with < 100 below LOD values: 66; 99; 101; 105; 146; 170; 180; 187; and D05. 21 PCB variables with > = 100 below LOD values: 126; 128; 156; 157; 167; 169; 172; 177; 178; 183; D01; D03; D04; D07; F03; F04; F05; F08; F10; TC2; and TCDD. One OCP variable with <100 below LOD values: p-p’-DDE and four OCP variables with > = 100 Below LOD values: trans-nonachlor, oxychlordane, p-p’-DDT, and Mirex.

The 36 PCBs were divided into Dioxin-Like (DL, n = 19) and Non-Dioxin-Like (NDL, n = 17) groups. Two composite exposure variables were created for these “total” variables by adding the 19 and 17 indicator variables described above. For the Total DL variable, the sum of the 19 indicator variables that ranged in value from 0 to 3 could result in a score ranging from 0 to 57. Total NDL ranged from 0 to 51. Once these sums were computed, individuals were divided into 4 exposure groups for each. The cutoff scores for the four groups for Total DL were: 0-10, 11-19, 20-34, and 35-57. For Total NDL, the cutoff scores were: 0-12, 13-23, 24-36, and 37-51. In summary, each individual had two additional indicator variables, for DL and NDL that ranged from 0 to 3, with 0 indicating the lowest level of exposure to a particular combination of compounds.

SAS PROC Surveyreg was used in all analyses to examine differences in blood measures across the four exposure groups for each of the 41 individual PCB/OCP compounds and for the two total compounds, controlling for gender and age. For each test of a dependent blood variable (e.g., white blood cell count, red blood cell count), gender, age and one of the 43 indicator variables were used as the independent variables. The PCB/OCP indicator variable was treated as a class variable, and the pdiff option was used to perform all six possible pairwise comparisons of blood means for the four levels of PCB/OCP exposure. Tests in which the p-value for the PCB/OCP predictor was less than 0.10 were followed by the six pairwise post hoc tests. The p-value for these tests was set at ≤ 0.05. Since all statistical tests were specified a priori, we followed recommendations to report p-values rather than adjust for multiple comparisons [29, 30].

To evaluate the influences of selected medical conditions on final results, regression analyses were repeated with three data subsets: 1) participants with self-reported cancer diagnosis removed, 2) participants with anemia treatment within the last 3 months removed, and 3) participants with autoimmune diseases, rheumatoid arthritis (RA) and type 1 diabetes mellitus (T1D, defined as diagnosed before age of 20 and use of insulin) by self-report, removed.

Correlations between individual DL and NDL PCBs were assessed using SAS PROC Surveyreg using one dependent variable and one independent variable and Pearson coefficients were calculated by taking the square root of R square for the regression.

## Results

**Figure 1:**
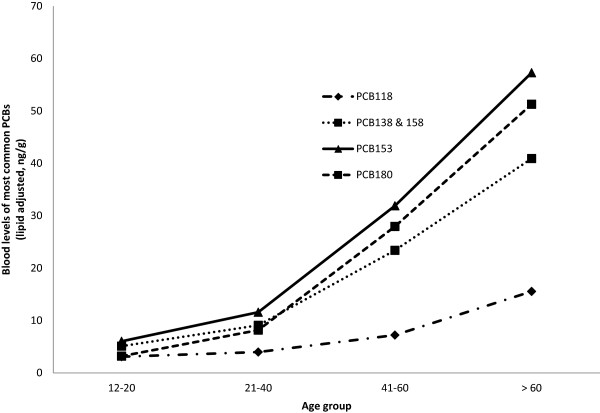
**Blood levels of most common PCBs (lipid adjusted) in the general population by age group (NHANES 2003-2004).**

**Figure 2:**
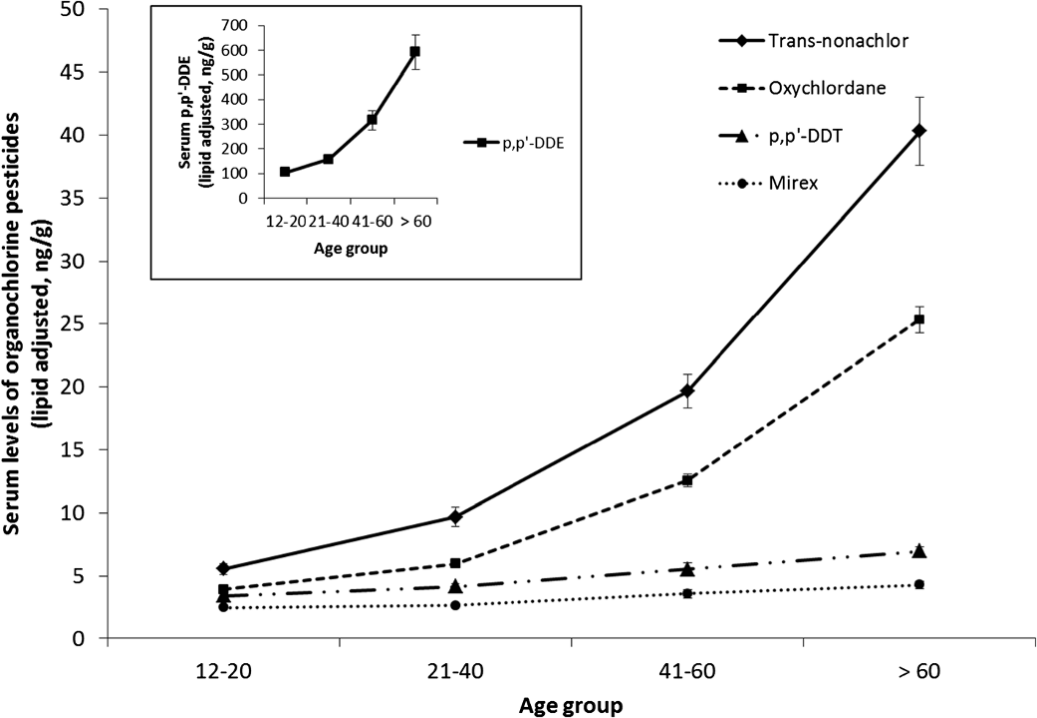
**Blood levels of organochlorine pesticides (lipid adjusted) in the general population by age group (NHANES 2003-2004).**

PCB and OCP levels increased with age and the highest levels were observed among those who were above 60 years old. Figures 1 and 2 present levels of most commonly quantified PCBs and selected organochlorine pesticides in serum samples from the NHANES population. Serum levels of PCBs (DL and NDL) and of organochlorine pesticides are positively associated with age and were highest among the oldest members of the population. When adjusted for age, we observed that most PCBs and OCPs in the 2003-2004 NHANES cycle were higher in males as compared to females (data not shown). A few exceptions where females had higher geometric mean (GM) values than males were for the DL PCB congeners: 105, 118, 167, D04, D05, 126, TCDD, and the following NDL PCB congeners: 28, 66, 74, and 99.

Correlations between DL PCBs and NDL PCBs varied (see Additional file 1). Pearson correlation coefficients with statistical significance (p < 0.05) ranged from 0.160 (between DO1 and PCB28) to 0.903 (between PCB118 and PCB74). Consistently, most dioxin like chemicals did not correlate with PCB101 and PCB52, and showed only weak correlations with PCB128 and PCB28. Polychlorinated dibenzofurans (such as F08 and F10) or coplanar polychlorinated biphenyls (such as TC2) showed fewer and weaker correlations with NDL PCBs in comparison to the other dioxin like compounds (see Additional file 1).

Tables 1 and 2 present selected blood parameters with statistically significant differences across PCB exposure groups in multiple comparison tests for total DL and NDL PCBs, respectively. Blood parameters presented consistent differences across exposure groups of individual DL and NDL PCBs (see Additional file 2). While we observed changes in the distribution (percentages) of blood cells, the absolute numbers of red and white blood cells decreased consistently as PCB levels increased. The decline of total count of white blood cells (WBC) was particularly consistent across exposure groups for all PCBs. For total DL and NDL groups, WBC counts were approximately 1,000,000 cells/ml lower in the highest exposure groups when compared to the lowest PCB group (Tables 1 and 2). For individual PCBs, the biggest differences in WBC counts were observed for the PCBs 178,180, 146, 172, 177, 170, 153, 146, and 187, all of which are NDL (see Additional file 2). Difference between the highest and lowest exposure groups reached 1,250,000 of WBC per ml of blood for PCB 178 (Additional file 2, lines 15661-15667). For most DL PCBs, even though a similar decline of WBC was observed at the highest exposure levels, the difference in total cell counts did not reach statistical significance (Additional file 2). Red blood cell counts (RBC), hematocrit, hemoglobin levels, and number of platelets declined as PCBs increased across groups of total NDL and DL (Tables 1 and 2) as well as for individual PCBs. RBC decline by exposure groups of total NDL PCBs did not reach statistical significance (70 million cells lower per ml of blood in the highest exposure group as compared to the lowest group of NDL PCBs). For individual PCBs the largest decline in RBC was observed for the two NDL PCBs178 and 177 (NDL PCBs), and for the DL PCBs156, 167, 169; 1,2,3,7,8-pncdd; 1,2,3,6,7,8-hxcdf; 1,2,3,4,6,7,8,9-ocdf. The difference in mean RBC number between the highest and lowest exposure groups of PCB 178 was 180 million cells per ml of blood. We also observed gradual decreases in levels of C-reactive protein as levels of DL and NDL PCBs increased, but comparisons were significant for NDL PCBs only (Table 2).

**Table 1:** **Geometric mean levels (and 95% Confidence Intervals) of complete blood count data by serum dioxin like (DL) PCB exposure groups (1 = lowest, 4 = highest)**

Blood count data	n	Total dioxin like (DL) PCBs			
		1	2	3	4
Segmented neutrophils number (10^6^ cells/mL)	1925	4.32 (4.04, 4.63)	4.06 (3.89, 4.23)	4.05 (3.94, 4.16)	3.69^*,#,†^ (3.54, 3.86)
Hematocrit (%)	1935	42.9 (42.3, 43.5)	42.9 (42.3, 43.6)	42.9 (42.6, 43.3)	42^*,#,†^ (41.5, 42.5)
Hemoglobin (g/dL)	1935	14.5 (14.3, 14.8)	14.6 (14.4, 14.8)	14.5 (14.4, 14.7)	14.2^*,#,†^ (14.1, 14.4)
Lymphocyte percent (%)	1925	28.1 (27, 29.2)	29.1 (28.4, 29.9)	28.5 (27.6, 29.4)	29.9^*,†^ (29.1, 30.7)
Mean cell hemoglobin (pg)	1935	30.4 (30, 30.8)	30.8^*^ (30.4, 31.1)	30.6 (30.4, 30.8)	30.4 (30.1, 30.8)
Monocyte percent (%)	1925	7.13 (6.76, 7.52)	7.37 (7.06, 7.69)	7.37 (7.17, 7.59)	7.82^*,†^ (7.56, 8.08)
Platelet count (10^6^ cells/mL)	1935	274 (268, 281)	257^*^ (249, 266)	263 (255, 271)	250^*,†^ (242, 257)
Red blood cell count (10^9^/mL)	1935	4.77 (4.71, 4.84)	4.74 (4.69, 4.8)	4.76 (4.72, 4.79)	4.67^*,†^ (4.62, 4.73)
White blood cell count (10^6^ cells/mL)	1935	7.36 (6.97, 7.78)	7.04 (6.77, 7.33)	6.95 (6.76, 7.15)	6.45^*,#,†^ (6.2, 6.72)

Comparisons are adjusted for age and gender. *p < 0.05 when compared to geometric mean of the first group; ^#^p < 0.05 when compared to geometric mean of the second group; ^†^p < 0.05 when compared to geometric mean of the third group.

**Table 2:** **Geometric mean levels (and 95% confidence intervals) of complete blood count data by serum non dioxin like (NDL) PCB exposure groups (1 = lowest, 4 = highest)**

Blood count data	n	Total non dioxin like (NDL) PCBs			
		1	2	3	4
Lymphocyte number (10^6^ cells/mL)	1889	2.09 (1.97, 2.22)	2.03 (1.94, 2.13)	1.96 (1.87, 2.04)	1.93^*^ (1.87, 1.99)
Segmented neutrophils number (10^6^ cells/mL)	1889	4.38 (4.1, 4.68)	4.2 (3.97, 4.44)	3.93^*^ (3.83, 4.03)	3.62^*,#,†^ (3.48, 3.77)
C-reactive protein (mg/dL)	1899	0.21 (0.181, 0.243)	0.163 (0.139, 0.191)	0.133^*^ (0.118, 0.151)	0.12^*,#^ (0.1, 0.144)
Hematocrit (%)	1899	42.4 (41.9, 43)	42.9 (42.5, 43.3)	43.1^*^ (42.6, 43.7)	42.2^#,†^ (41.7, 42.6)
Hemoglobin (g/dL)	1899	14.4 (14.2, 14.6)	14.6 (14.4, 14.7)	14.6 (14.4, 14.8)	14.2^*,#,†^ (14.1, 14.4)
Lymphocyte percent (%)	1889	28.1 (27.2, 29)	28.3 (27.2, 29.5)	28.9 (28.2, 29.6)	30.1^*,†^ (29.1, 31.2)
Mean cell hemoglobin concentration (g/dL)	1899	34 (33.8, 34.2)	34 (33.8, 34.2)	33.9^#^ (33.7, 34.1)	33.8^*,#^ (33.6, 33.9)
Mean cell hemoglobin (pg)	1899	30.4 (30.1, 30.6)	30.7* (30.4, 31.1)	30.7 (30.5, 30.9)	30.4 (30.1, 30.7)
Mean cell volume (fL)	1899	89.4 (88.1, 90.7)	90.4^*^ (89.1, 91.7)	90.6^*^ (89.4, 91.9)	90 (88.6, 91.5)
Segmented neutrophils percent (%)	1889	58.9 (57.9, 59.8)	58.5 (57.5, 59.6)	57.9 (56.9, 58.9)	56.4^*,#,†^ (55.3, 57.5)
Platelet count SI (10^6^ cells/mL)	1899	270 (263, 278)	265 (259, 270)	261 (252, 269)	244^*,#^ (232, 256)
White blood cell count (10^6^ cells/mL)	1899	7.46 (7.03, 7.91)	7.17 (6.83, 7.52)	6.78^*^ (6.59, 6.98)	6.4^*,#,†^ (6.23, 6.57)

Comparisons are adjusted for age and gender. *p < 0.05 when compared to geometric mean of the first group; ^#^p < 0.05 when compared to geometric mean of the second group; ^†^p < 0.05 when compared to geometric mean of the third group.

Tables 3 and 4 present blood biochemistry data of statistically significant changes across PCB qroups in multiple comparison tests for total DL and NDL PCBs, respectively. The most consistent patterns for blood biochemistry data across PCB exposure groups were observed for the liver enzymes ALT, AST, and GGT which were respectively 1.06, 1.04, and 1.12-fold higher in the highest group of DL PCBs when compared to the lowest exposure group (Table 3). Levels of alkaline phosphatase on the other hand dropped as levels of PCBs increased (Tables 3 and 4). Total serum bilirubin levels increased as PCB exposures increased (Tables 3 and 4).

**Table 3:** **Geometric mean levels (and 95% confidence intervals) of blood biochemistry data by serum dioxin like (DL) PCB groups exposure groups (1 = lowest, 4 = highest)**

Blood marker	n	Total dioxin like (DL) PCBs			
		1	2	3	4
Albumin (g/L)	1935	42.1 (41.7, 42.5)	42.8^*^ (42.4, 43.3)	43.2^*^ (42.8, 43.7)	43^*^, (42.6, 43.5)
Alanine aminotransferase ALT (U/L)	1933	21.5 (20.2, 23)	21.1 (20.3, 21.9)	23.2^#^ (22.3, 24.2)	22.9^#^ (21.7, 24.2)
Aspartate aminotransferase AST (U/L)	1933	23.4 (22.6, 24.3)	22.8 (22, 23.7)	24.1^#^ (23.3, 24.8)	24.4^#^ (23.4, 25.5)
Alkaline phosphotase (U/L)	1935	79.6 (74.7, 84.9)	68.8^*^ (64.8, 72.9)	65.9^*^ (63.6, 68.3)	73.3^*,†^ (69.7, 77.1)
Blood urea nitrogen (mmol/L)	1935	3.72 (3.5, 3.95)	3.79 (3.59, 4.01)	3.91 (3.73, 4.1)	4.14^*,†^ (3.97, 4.32)
Gamma glutamyl transferase GGT (U/L)	1934	18.8 (17.1, 20.6)	18.2 (17.2, 19.2)	20.2^#^ (19.2, 21.2)	21.1^#^ (19.2, 23.2)
Phosphorus (mmol/L)	1934	1.27 (1.25, 1.29)	1.23^*^ (1.22, 1.25)	1.24^*^ (1.22, 1.26)	1.26 (1.24, 1.28)
Bilirubin, total (μmol/L)	1934	11.3 (10.5, 12.2)	12.6^*^ (12, 13.3)	12.6^*^ (12, 13.2)	13.1^*^ (12.5, 13.6)
Creatinine (μmol/L)	1935	75.4 (72.9, 77.9)	75.7 (74.2, 77.1)	74.7 (73.1, 76.3)	70.5^#,†^ (67.6, 73.4)
Potassium (mmol/L)	1934	4.05 (4.01, 4.08)	4^*^ (3.96, 4.04)	3.96^*^ (3.91, 4.01)	3.93^*,#^ (3.89, 3.97)
Globulin (g/L)	1934	29.4 (28.9, 29.9)	28.9 (28.3, 29.6)	28.7 (28.2, 29.3)	28.5^*^ (28, 29.1)

Comparisons are adjusted for age and gender. *p < 0.05 when compared to geometric mean of the first group; ^#^p < 0.05 when compared to geometric mean of the second group; ^†^p < 0.05 when compared to geometric mean of the third group.

**Table 4:** **Geometric mean levels (and 95% confidence intervals) of blood biochemistry data by serum non dioxin like (NDL) PCB exposure groups (1 = lowest, 4 = highest)**

Blood marker	n	Total non dioxin like (NDL) PCBs			
		1	2	3	4
Alanine aminotransferase ALT (U/L)	1897	22.2 (20.6, 23.8)	20.9 (19.9, 21.9)	23.1^#^ (22.4, 23.8)	22.6^#^ (21.6, 23.8)
Aspartate aminotransferase AST (U/L)	1897	23.6 (22.7, 24.5)	22.8 (22, 23.6)	23.9^#^ (23.1, 24.7)	24.7^#^ (24, 25.4)
Alkaline phosphotase (U/L)	1899	79.8 (76.1, 83.7)	66.7^*^ (62.5, 71.2)	67.9^*^ (65.3, 70.6)	71.4 (66.2, 76.9)
Bicarbonate (mmol/L)	1899	24.3 (23.9, 24.7)	24.8^*^ (24.4, 25.1)	24.7^*^ (24.5, 25)	24.8^*^ (24.4, 25.1)
Iron (μmol/L)	1897	13.7 (13.2, 14.3)	14.2 (13.6, 14.7)	15^*,#^ (14.6, 15.5)	14.6^*^ (13.9, 15.3)
Lactate dehydrogenase LDH (U/L)	1897	129 (125, 133)	124^*^ (121, 126)	124^*^ (121, 128)	122^*^ (119, 126)
Bilirubin, total (μmol/L)	1898	11.6 (10.9, 12.3)	12.8^*^ (12.2, 13.5)	12.7^*^ (12.1, 13.3)	12.6^*^ (12.2, 13)
Uric acid (μmol/L)	1898	321 (308, 334)	309 (303, 315)	302^*^ (294, 309)	298^*^ (287, 308)
Creatinine (μmol/L)	1899	76 (74.1, 78)	76.4 (75.1, 77.7)	73.7^#^ (72.4, 75.1)	70.7^*,#,†^ (68.3, 73.1)
Sodium (mmol/L)	1898	139.3 (138.7, 139.9)	139.0^*^ (138.4, 139.6)	139.3 (138.7, 139.9)	138.9 (138.2, 139.5)
Potassium (mmol/L)	1898	4.04 (4, 4.08)	4.01 (3.97, 4.06)	3.97^*,#^ (3.93, 4.01)	3.9^*,#,†^ (3.87, 3.94)
Chloride (mmol/L)	1899	103.9 (103.1, 104.7)	103.5 (102.8, 104.2)	103.7 (103.0, 104.4)	103.3† (102.6, 104)
Osmolality (mmol/kg)	1898	277.8 (276.5, 279.1)	276.7^*^ (275.4, 278)	277.3 (276, 278.6)	276.7^*^ (275.3, 278)
Globulin (g/L)	1898	29.4 (28.9, 30)	28.7^*^ (28.1, 29.3)	28.4^*^ (27.7, 29)	29.2 (28.3, 30)

Comparisons are adjusted for age and gender. *p < 0.05 when compared to geometric mean of the first group; ^#^p < 0.05 when compared to geometric mean of the second group; ^†^p < 0.05 when compared to geometric mean of the third group.

While changes in blood count data were consistent for different PCB types, the measurements varied considerably when analyses were repeated for different types of organochlorine pesticides (Table 5). Serum Mirex: Lymphocyte number and segmented neutrophil numbers appeared to be lower in the highest exposure groups, while the percentages of basophils and monocytes appeared to be higher. Mean cell total hemoglobin and hemoglobin concentrations were lower in the highest exposure group, while WBC count was significantly lower in this group. Serum p-p’-DDE and p-p’-DDT: Blood count results presented a similar pattern across exposure groups described for both pesticides. As serum levels of pesticides increased, lymphocyte % values increased. Values of segmented neutrophil %, mean cell total hemoglobin and mean cell volume on the other hand presented a decrease. For p-p’-DDT we also observed an apparent decrease in segmented neutrophil numbers and in mean cell hemoglobin concentration. No significant changes were noticed in total cell numbers across groups of p-p’-DDE levels (data not shown). For levels of p-p’-DDT, however; higher numbers of lymphocytes and lower numbers of segmented neutrophils were observed at the highest exposure groups (Table 5).

**Table 5:** **Geometric mean levels (and 95% confidence intervals) of selected complete blood count data by serum organochlorine pesticide exposure groups (1 = lowest, 4 = highest)**

Blood marker	n	Mirex			
		1	2	3	4
Lymphocyte number (10^6^ cells/mL)	1940	2.05 (1.99, 2.1)	2.25^*^ (2.16, 2.34)	2.05 (1.88, 2.22)	2^#^ (1.9, 2.11)
Segmented neutrophils number (10^6^ cells/mL)	1940	4.14 (4.02, 4.26)	4.15 (3.96, 4.35)	3.79 (3.49, 4.12)	3.85^*^ (3.66, 4.05)
White blood cell count (10^6^ cells/mL)	1951	7.13(6.98, 7.3)	7.32 (7.07, 7.57)	6.81 (6.4, 7.24)	6.79^*,#^ (6.55, 7.03)
		**p,p’-DDT**			
Lymphocyte number (10^6^ cells/mL)	1954	1.96 (1.88, 2.04)	2.13^*^ (2.03, 2.24)	2.08 (2.01, 2.15)	2.11^*^ (2.02, 2.21)
Segmented neutrophils number (10^6^ cells/mL)	1954	4.2 (4.03, 4.36)	4.08 (3.92, 4.26)	4.05 (3.87, 4.25)	3.81^*,#^ (3.69, 3.93)
		**Trans-nonachlor**			
Lymphocyte number (10^6^ cells/mL)	1944	1.91 (1.82, 2.01)	1.95 (1.88, 2.02)	2.1^*^ (1.99, 2.21)	2.19^*,#,†^ (2.11, 2.26)
Segmented neutrophils number (10^6^ cells/mL)	1944	3.68 (3.47, 3.89)	4.08^*^ (3.94, 4.23)	4.12^*^ (3.94, 4.31)	4.08^*^ (3.9, 4.26)
White blood cell count (10^6^ cells/mL)	1955	6.5 (6.23, 6.78)	6.97^*^ (6.78, 7.16)	7.15^*^ (6.9, 7.42)	7.24^*^ (7.03, 7.46)
		**Oxychlordane**			
Lymphocyte number (10^6^ cells/mL)	1967	1.92 (1.83, 2.02)	2.07^*^ (2.01, 2.14)	2.07^*^ (1.99, 2.17)	2.18^*^ (2.06, 2.32)
Segmented neutrophils number (10^6^ cells/mL)	1967	3.85 (3.67, 4.04)	4.31^*^ (4.18, 4.44)	3.99^#^ (3.83, 4.17)	4.08 (3.86, 4.3)
White blood cell count (10^6^ cells/mL)	1978	6.68 (6.42, 6.94)	7.36^*^ (7.22, 7.51)	6.98^#^ (6.76, 7.21)	7.24^*^ (6.95, 7.54)

Comparisons are adjusted for age and gender. *p < 0.05 when compared to geometric mean of the first group; ^#^p < 0.05 when compared to geometric mean of the second group; ^†^p < 0.05 when compared to geometric mean of the third group.

For trans-nonachlor and oxychlordane: Lymphocyte number and segmented neutrophils numbers increased as the pesticide levels increased. In contrast to all the other chlorinated chemicals, we observed an increase in WBC numbers as the levels of trans-nonachlor and oxychlordane increased (Table 5).

Similar to CBC counts, results of blood biochemistry data varied by the type of organochlorine pesticide (Table 6). A few of the analytes were significantly different across categories of pesticide biomarkers. Those differences were as follows:

**Table 6:** **Geometric mean levels (and 95% confidence intervals) of liver enzymes by serum organochlorine pesticide exposure groups (1 = lowest, 4 = highest)**

Blood marker		Mirex			
	n	1	2	3	4
Aspartate aminotransferase AST (U/L)	1951	23.2 (22.7, 23.6)	23.7 (22.3, 25.3)	24.3 (23.2, 25.5)	25^*^ (23.6, 26.5)
Gamma glutamyl transferase GGT (U/L)	1951	18.8 (18.2, 19.4)	22.2^*^ (20.2, 24.4)	22^*^ (19, 25.3)	19.7 (16.6, 23.2)
		**p,p’-DDE**			
Alanine aminotransferase ALT (U/L)	1956	20.9 (20, 21.8)	22.9 (21.3, 24.5)	22.7^*^ (21.7, 23.7)	22.5^*^ (21.4, 23.6)
Gamma glutamyl transferase GGT (U/L)	1956	19 (18, 20)	21.1^*^ (19.4, 22.9)	19.7 (17.8, 21.7)	18.9 (17.2, 20.7)
		**p,p’-DDT**			
Alanine aminotransferase ALT (U/L)	1965	20.4 (19.5, 21.3)	23.3^*^ (22, 24.7)	22.8^*^ (21.6, 24)	22.6^*^ (21.6, 23.7)
Aspartate aminotransferase AST (U/L)	1965	22.6 (22, 23.2)	24.1^*^ (23.1, 25.3)	23.8 (22.8, 24.7)	24^*^ (23.3, 24.7)
Gamma glutamyl transferase GGT (U/L)	1965	18.1 (17, 19.2)	20.7^*^ (18.9, 22.6)	20.3^*^ (18.7, 22)	19.8 (17.8, 21.9)
		**Trans-nonachlor**			
Alanine aminotransferase ALT (U/L)	1955	19.3 (17.9, 20.8)	21 (20.2, 21.8)	23.1^*,#^ (22.1, 24.1)	23.3^*,#^ (21.9, 24.9)
Gamma glutamyl transferase (U/L)	1955	15.4 (14.1, 16.9)	19^*^ (18, 20)	20.4^*^ (19, 21.9)	21.3^*^ (18.9, 24)
		**Oxychlordane**			
Alanine aminotransferase ALT (U/L)	1978	20.1 (19.2, 21.1)	22^*^ (20.9, 23.1)	23.7^*^ (22.5, 25)	22^†^ (20.7, 23.4)
Gamma glutamyl transferase GGT (U/L)	1978	16.1 (14.6, 17.9)	19.9^*^ (18.6, 21.3)	21.4^*^ (19.6, 23.3)	20.5^*^ (18.2, 23.2)

Comparisons are adjusted for age and gender. *p < 0.05 when compared to geometric mean of the first group; ^#^p < 0.05 when compared to geometric mean of the second group; ^†^p < 0.05 when compared to geometric mean of the third group.

Serum Mirex: Measures of AST and GGT increased as levels of pesticides increased. Serum glucose appeared to be higher in the second exposure group, but was at lower concentrations for the third and fourth (highest) exposure groups.

Serum p-p’-DDE and p-p’-DDT: As levels of serum p-p’-DDT increased so did the levels of serum ALT, AST, GGT (Table 6) and glucose (data not shown). For p-p’-DDE, we observed increases in levels of ALT, glucose, and total bilirubin at the highest exposure groups (p < 0.05 when compared to the lowest group, data not shown). Levels of GGT were also higher in the second and third exposure groups when compared to the lowest exposure group (Table 6).

For trans-nonachlor and oxychlordane: Levels of serum ALT and GGT increased as exposure to these chemicals increased (Table 6). We also observed higher levels of blood glucose at the highest exposure groups of both chemicals when compared to the lowest group (p < 0.05, data not shown).

Regression analyses were repeated in three subsets of the data to evaluate the influence of cancer, possible autoimmune disease (Type 1D and RA), and anemia. Statistically significant results of these analyses are presented in supplementary files (Additional files 3, 4, 5, 6, 7 and 8). Overall, changes in blood parameters showed a consistent pattern for all exposure types in these different subset analyses. Table 7 presents a comparison of white blood cell count (by DL and NDL PCB exposure groups) and red blood cell count (by DL PCBs) results of subset analyses.

**Table 7:** **Geometric mean levels (and 95% Confidence Intervals) of white blood cell and red blood cell counts by serum PCB groups (1 = lowest, 4 = highest)**

**Data set used**	**n**	**White blood cell count (10** ^**6**^ **cells/mL) by total NDL PCB groups**			
		**1**	**2**	**3**	**4**
**All individuals**	1899	7.46 (7.03, 7.91)	7.17 (6.83, 7.52)	6.78^*^ (6.59, 6.98)	6.4^*,#,†^ (6.23, 6.57)
**Anemia removed**	1754	7.42 (7.0, 7.86)	7.11 (6.81, 7.42)	6.843^*^ (6.64, 7.05)	6.42^*,#,†^ (6.17, 6.69)
**Cancer removed**	1729	7.38 (6.97, 7.82)	7.08 (6.79, 7.39)	6.86 (6.65, 7.07)	6.47^*,#,†^ (6.22, 6.73)
**T1D and RA removed**	1820	7.44 (7.02, 7.88)	7.12 (6.82, 7.43)	6.84^*^ (6.63, 7.05)	6.45^*,#,†^ (6.22, 6.69)
**Data set used**	**n**	**White blood cell count (10** ^**6**^ **cells/mL) by total DL PCB groups**			
		**1**	**2**	**3**	**4**
**All individuals**	1935	7.36 (6.97, 7.78)	7.04 (6.77, 7.33)	6.95 (6.76, 7.15)	6.45^*,#,†^ (6.2, 6.72)
**Anemia removed**	1786	7.39 (6.99, 7.81)	7.026 (6.78, 7.28)	6.92 (6.7, 7.16)	6.53^*,#,†^ (6.29, 6.78)
**Cancer removed**	1763	7.44 (7.01, 7.89)	7.03 (6.79, 7.26)	6.92^*^ (6.72, 7.13)	6.47^*,#,†^ (6.18, 6.77)
**T1D and RA removed**	1853	7.41 (7.04, 7.80)	7.04 (6.78, 7.3)	6.96 (6.75, 7.18)	6.48^*,#,†^ (6.23, 6.74)
**Data set used**	**n**	**Red blood cell counts (10** ^**9**^ **cells/mL) by total DL PCB groups**			
		**1**	**2**	**3**	**4**
**All individuals**	1935	4.77 (4.71, 4.84)	4.74 (4.69, 4.8)	4.76 (4.72, 4.79)	4.67^*,†^ (4.62, 4.73)
**Anemia removed**	1786	4.78 (4.72, 4.85)	4.77 (4.71, 4.83)	4.78 (4.74, 4.82)	4.69^†^ (4.64, 4.74)
**Cancer removed**	1763	4.79 (4.72, 4.85)	4.76 (4.71, 4.82)	4.78 (4.74, 4.81)	4.67^*,#,†^ (4.61, 4.73)
**T1D and RA removed**	1853	4.77 (4.71, 4.84)	4.76 (4.70, 4.82)	4.77 (4.73, 4.80)	4.68^†^ (4.62, 4.73)

Comparisons are adjusted for age and gender. *p < 0.05 when compared to geometric mean of the first group; ^#^p < 0.05 when compared to geometric mean of the second group; ^†^p < 0.05 when compared to geometric mean of the third group.

## Discussion

Despite a manufacturing ban over three decades ago, measurable amounts of PCBs and chlorinated pesticides are present in serum samples obtained recently from the general population. Highest serum levels were observed in the elderly which is consistent with previous reports [31–33]. The age difference may reflect bioaccumulation of these chemicals over the years, the reduced exposures faced by the younger generation, or both.

We report possible associations between selected chlorinated compounds and common blood measures involving immune cells and blood biochemistry. Overall, we observed lower levels of total WBC number, RBC count, hemoglobin and hematocrit measures in the group with the highest serum PCB levels. To our knowledge this is the first study to report an association between PCBs and WBC and RBC in the general population. Daniel et al [34] examined cellular and humoral immune response among individuals with occupational exposures to PCBs. Overall, patients with higher levels of these persistent chemicals in their blood had lower levels of T lymphocyte counts, lower INF-gamma levels, and higher GGT plasma levels [34]. Somewhat consistent findings have been reported for cases of prenatal exposure to PCBs, in which increased risk of early childhood infections was observed in different cohorts across the world [9, 35–37]. Among the Inuit population, who are known to have higher levels of exposures to organochlorines [38], umbilical cord blood levels of PCBs were associated with increased incidence of acute otitis media and lower respiratory tract infections during the first five years of life [36]. Similarly, prenatal exposure to PCBs has been linked to increased risks of developing infections within the first three years of life in a Norwegian mother and child cohort [35]. Accidental PCB poisoning through consumption of contaminated rice bran oil in Taiwan in 1979 showed marked immunosuppressive effects among those affected, with many patients suffering from a wide range of infections [20]. Laboratory findings included decreased IgM and IgA levels, decreased percentage of total T cells and helper T-cells (CD4), and suppressed antigen recall response such as delayed hypersensitivity and tuberculin skin test [20]. Conflicting results were observed in samples collected one week after an accidental spill in New York, where exposed workers had higher mean RBC counts, hemoglobin and hematocrit measurements than matched controls [39]. Even though blood levels of PCBs were not significantly different across exposure groups, the authors speculated that an increase in RBC may be due to an acute response to an increased oxygen demand among the exposed [39].

While the measures of WBC and RBC in this study were within normal clinical ranges, the consistency of findings for different PCBs is noteworthy and needs to be considered in the context of chronic low level exposures. Depression of the humoral and cellular immune response by PCBs, including reduced numbers of WBC, has been previously reported in animals, such as guinea pigs and rats [12, 40, 41]. Studies in monkeys reported decreased antibody (IgG and IgM) production against sheep red blood cells [18, 42, 43], and alterations in T-cell subsets such as increased T-suppressor cells (CD8) and decreased T-helper cells (CD4) [12]. Our groups with the highest and lowest levels of serum DL PCBs had a difference of 100 million RBC per ml of blood. This difference is higher than the decline (60 million RBC/ml) observed in rhesus monkeys fed with 40 μg/kg arochlor1254 for 34 months [14]. Other findings from this experimental study are consistent with our results, showing decreased levels of RBC, hematocrit, mean platelet volume, and WBC [14]. Consistently, anemia, elevated liver enzymes, and an inverse association between functional immune assays (T-lymphocyte proliferation, neutrophil and monocyte phagocytosis) and tissue levels of PCBs were among the effects observed among bottlenose dolphins exposed to PCBs [44].

Results for the OCPs were not as consistent as those observed in PCBs. WBC count measures varied by the type of the pesticides. WBC were lowest at the highest exposure of Mirex, were similar across groups of p-p’-DDT or p-p’-DDE values, and were higher at the highest exposure qroups of trans-nonachlor and its metabolite oxychlordane. While there are no comparable human studies, our results are consistent with previous experimental findings. In a study with Sprague-Dawley rats treated with trans-nonachlor for 28 days, immunotoxicologic effects involved a significant increase in the absolute number of WBC as dose of trans-nonachlor increased [45]. The varying effects of OCPs may be linked to their different chemical structures and needs to be further examined.

While previous studies reported possible effects of PCBs on the immune system, specific mechanisms are not known. Aryl hydrocarbon (Ah) receptor involvement has been proposed for dioxin-like PCBs due to their structural similarities with aromatic hydrocarbons [12]. In fact, most congener specific studies focused on dioxin-like PCBs and suggested that PCB effects on the immune system are positively associated with the number of chlorine atoms in the molecule [12]. There is some evidence that chlorine substitution affects PCB affinity for the Ah receptor and is associated with severe suppression of the antibody response in mice [46]. Most animal studies were conducted using only specific PCB congeners ignoring potential interactions among various PCBs, or with other chemicals in the environment. Our observations are within normal clinical parameters, but the direction of findings is consistent with previous experimental studies. Our results also suggest that PCBs may impact numbers of WBC and RBC in the general population and that this effect is not limited to DL PCBs. In fact, we observed the biggest decline in WBC for NDL PCBs. This suggests that for effects on WBCs mechanisms other than the Ah receptor may be involved and that general application of toxic equivalency factors based on dioxin may lead to erroneous conclusions.

PCBs are considered hepatotoxic based mostly on animal studies. Increased liver weight, enzyme induction, increased levels of serum liver enzymes have been documented in animals and usually in connection with DL PCBs [1, 47]. PCBs, particularly those with higher number of chlorine, have also been linked to liver cancer in female rats [47–49]. Our most consistent findings for blood biochemistry measures were the higher levels of the liver enzymes (ALT, AST, and GGT) at the highest exposure groups of PCBs and organochlorine pesticides. A positive association between PCBs and ALT was reported previously in the general population [50, 51]. Here we expand these findings to include AST and GGT. Findings for GGT are particularly noteworthy since recent studies have pointed to the usefulness of this enzyme as an early and sensitive marker of xenobiotics exposure, even if the levels are observed within normal clinical parameters [52, 53]. In this study, participants in the highest exposure groups of DL PCBs and OCPs had the highest levels of serum GGT. Thus, it is possible that the higher levels of liver enzymes might be indicators of subtle effects in liver function due to environmental insult. It is important to also note that factors potentially altering liver enzyme levels, such as alcohol consumption, diseases of the liver or bile ducts, or use of medication were not considered in our analyses and limit our ability to draw conclusions.

Several limitations have to be considered when interpreting our study findings. First, the nature of cross-sectional study design limits any causality assessment and assumptions of temporality. Thus, our results cannot be used to draw definite conclusions on associations between chlorinated chemical exposures and blood markers. Yet the consistency of the observed associations support the generation of new hypotheses and justification of future studies. We have also not been able to assess any functional markers of the immune system, such as specific antibody responses to antigens. Second, we have identified age and gender as the main confounders altering our outcomes, but we have not considered the effects of individual factors that may be important predictors, such as medical history, alcohol consumption, dietary exposures, or use of medication. However, we evaluated the influence of cancer diagnosis, anemia treatment and possible autoimmune diseases in separate analyses of three subsets and observed consistent findings. Third, a major limitation in environmental epidemiology is the multitude of exposures in real life situations. We have only analyzed biomarkers of a few chemicals. Yet, simultaneous exposures to other chemicals may have impacted our results. And finally, due to the variation of analytical detection limits, we limited our data to the NHANES 2003-2004 cycle, which had the lowest detection limits. This resulted in a low samples size for some of the analyses.

## Conclusion

Our exploratory analyses suggest possible associations between serum levels of PCBs and OCPs and common blood parameters such as WBC, RBC numbers or liver enzymes in the general population. While several limitations in our study design prevent us from reaching final conclusions, our findings are consistent with previous animal studies and reports of accidental exposures in humans. All of the associations we observed are within normal clinical ranges. But considering the consistency of the effects and the fact that we have not evaluated potential combined effects of other pollutants, these associations are noteworthy and may reflect early effects in the general population. Toxicities of PCBs are usually reported by toxic equivalency factors (TEFs) that are based on dioxin like structures and aryl hydrocarbon receptor involvement. However, some of the largest differences observed in this study were across groups of NDLs. Thus, routine applications of TEFs to assess the effects of PCBs may not adequately represent the effects of NDLs in the mixture.

## Electronic supplementary material

Additional file 1: **Correlations among lipid adjusted levels of DL and NDL PCBs.** (XLSX 29 KB)

Additional file 2: **Regression analyses of individual PCBs.** (XLSX 2 MB)

Additional file 3: **Multiple comparisons of blood parameters across groups of DL and NDL PCBs (individuals with anemia removed).** (XLSX 19 KB)

Additional file 4: **Multiple comparisons of blood parameters across groups of OCPs (individuals with anemia removed).** (XLSX 53 KB)

Additional file 5: **Multiple comparisons of blood parameters across groups of DL and NDL PCBs (individuals with cancer removed).** (XLSX 52 KB)

Additional file 6: **Multiple comparison of blood parameters across groups of OCPs (individuals with cancer removed).** (XLSX 49 KB)

Additional file 7: **Multiple comparisons of blood parameters across groups of PCBs (individuals with T1D and RA removed).** (XLSX 20 KB)

Additional file 8: **Multiple comparisons of blood parameters across groups of OCPs (individuals with T1D and RA removed).** (XLSX 27 KB)

## Abbreviations

ALT: Alanine aminotransferase
AST: Aspartate aminotransferase
CBC: complete blood count
DL: Dioxin Like
DO1: 1,2,3,7,8-pentachlorodibenzo-p-dioxin
DO3: 1,2,3,6,7,8-hexachlorodibenzo-p-dioxin
DO4: 1,2,3,7,8,9- hexachlorodibenzo-p-dioxin
DO5: 1,2,3,4,6,7,8-heptachlorodibenzo-p-dioxin
DO7: 1,2,3,4,6,7,8,9-octachlorodibenzo-p-dioxin
FO3: 2,3,4,7,8-pentachlorodibenzofuran
FO4: 1,2,3,4,7,8-hexachlorodibenzofuran
FO5: 1,2,3,6,7,8-hexachlorodibenzofuran
FO8: 1,2,3,4,6,7,8-heptachlorodibenzofuran
F10: 1,2,3,4,6,7,8,9-octachlorodibenzofuran
GGT: Gamma glutamyl transferase
GM: Geometric mean
LDH: Lactate dehydrogenase
LOD: Limit of detection
NDL: Non Dioxin Like
NHANES: National Health and Nutrition Examination Survey
OCPs: Organochlorine pesticides
PCBs: Polychlorinated biphenyls
RBC: Red blood cells
RA: Rheumatoid arthritis
TC2: 3,4,4',5-tetrachlorobiphenyl
TCDD: 2,3,7,8-tetrachlorodibenzo-p-dioxin
T1D: Type 1 diabetes mellitus
WBC: White blood cells.
